# Genome Sequence of *Arthrobacter* Phage NathanVaag

**DOI:** 10.1128/mra.00940-22

**Published:** 2022-10-17

**Authors:** Rebecca A. Chong, Charlie M. Cosse, Viviana Gaytan, Dylan S. Green, Cade J. Kane, Kirsten A. Kasal, Zarek K. Kon, Rosa E. Maxwell, Vlas Olaru, Tuan D. Pham, MeiLin F. Precourt, Joseph R. Romero, Wellington J. Rothschild, Shantelle N. Sales, Troy D. Sensano, Wesley E. Simko, Inugiksuq J. Smith, Mika A. Toor, Adelaide R. Wilson, Megan L. Porter

**Affiliations:** a School of Life Sciences, University of Hawai`i at Mānoa, Honolulu, Hawai`i, USA; Portland State University

## Abstract

We report the genome sequence of bacteriophage NathanVaag, an actinobacteriophage isolated from soil in El Paso, Texas, that infects *Arthrobacter* sp. strain ATCC 21022. The 49,645-bp genome contains 73 predicted protein-coding genes. Based on gene content similarity to phages in the Actinobacteriophage Database, NathanVaag is assigned to phage cluster AO1.

## ANNOUNCEMENT

Understanding the molecular evolution of bacteriophages is critical to finding novel medical solutions for antibiotic resistance in bacteria, as well as for diverse applications in agricultural and biotechnological settings. Here, we present the genome sequence of a novel actinobacteriophage, NathanVaag, that infects the soil bacterium *Arthrobacter* sp. strain ATCC 21022 and that represents an additional sequenced actinobacteriophage genome isolated in Texas through the Science Education Alliance-Phage Hunters Advancing Genomics and Evolutionary Science (SEA-PHAGES) program at the University of Texas at El Paso (UTEP) (El Paso, Texas) and annotated through the SEA-PHAGES program at the University of Hawai`i at Mānoa (Honolulu, Hawai`i) (1, 2).

NathanVaag was isolated and purified in 2019 from a surface soil sample collected at UTEP (coordinates: 31.768794N, 106.505322W) using standard methods (3). Briefly, the soil sample was washed in peptone-yeast extract-calcium (PYCa) medium, and the filtered wash (0.22 μm) was inoculated with *Arthrobacter* sp. strain ATCC 21022. After incubation with shaking for 48 h at 28°C, the mixture was refiltered and plated in top agar with *Arthrobacter* sp. A single clear plaque was selected and isolated by enrichment following multiple rounds of plaque purification by plating in top agar with *Arthrobacter* sp. at 28°C. After phage particle purification, a high-titer lysate was generated from webbed plates, and genomic DNA was extracted using a Wizard DNA extraction kit (Promega). Genomic DNA was prepared for sequencing using the NEBNext Ultra II library kit and sequenced on an Illumina MiSeq instrument (v3 reagents), resulting in 167-fold coverage from 61,215 single-end 150-bp reads. Raw reads were assembled in Newbler v2.9 (Roche) using default parameters, resulting in a single genomic contig. Genome completeness and accuracy and phage genomic termini were verified using Consed v29 as described previously (4–6). The complete genome sequence of NathanVaag was circularly permuted and is 49,645 bp in length, with a G+C content of 63.6%.

The genome was autoannotated using DNA Master v5.23.6 build 2701 (http://cobamide2.bio.pitt.edu/computer.htm) embedded with Glimmer v3 (7) and GeneMark (8). Start sites from the autoannotation were manually refined using the Phage Evidence Collection and Annotation Network (PECAAN) (http://pecaan.kbrinsgd.org), Phamerator (9), and Starterator (http://phages.wustl.edu/starterator). Potential functions for predicted protein-coding genes were assigned based on top hits for searches using NCBI BLASTp (nonredundant database) (10) and HHpred (PDB mmcif70, NCBI conserved domain, SCOPe70, and Pfam databases) (11), and putative membrane proteins were identified using TMHMM v2.0 (https://services.healthtech.dtu.dk/service.php?DeepTMHMM). Default parameters were used for all software. NathanVaag is predicted to have 73 protein-coding genes, of which 36 genes (49%) have a putative function assigned and the remaining 37 genes (51%) encode hypothetical proteins with unknown functions. No tRNAs were identified using ARAGORN v1.2.38 (12) and tRNAscan-SE v2.0 (13). Of the 73 genes, all except the rightmost six genes are transcribed rightward. Based on gene content similarity of at least 35% to phages within the Actinobacteriophage Database (PhagesDB) (14), NathanVaag can be assigned to the AO1 subcluster of cluster AO actinobacteriophages (15). Similar to other AO1 phages, NathanVaag contains several genes with putative DNA cleavage functions (e.g., RecE-like exonuclease and RusA-like resolvase) and is predicted to be a lytic phage with no identifiable immunity repressor or integrase functions.

### Data availability.

The complete genome sequence of the actinobacteriophage NathanVaag has been deposited in GenBank with accession number ON970601, BioProject accession number PRJNA488469, and SRA accession number SRX14483220.
